# Prognostic significance of USP33 in advanced colorectal cancer patients: new insights into β-arrestin-dependent ERK signaling

**DOI:** 10.18632/oncotarget.13219

**Published:** 2016-11-08

**Authors:** Hongda Liu, Qun Zhang, Kangshuai Li, Zheng Gong, Zhaochen Liu, Yunfei Xu, Mary Hannah Swaney, Kunhong Xiao, Yuxin Chen

**Affiliations:** ^1^ Department of General Surgery, Qilu Hospital Affiliated to Shandong University, Jinan, Shandong 250012, China; ^2^ Department of Biochemistry and Molecular Biology, School of Medicine, Shandong University, Jinan, Shandong 250012, China; ^3^ Department of Pharmacology and Chemical Biology, School of Medicine, University of Pittsburgh, Pittsburgh, Pennsylvania 15261, USA; ^4^ Department of Respiratory Medicine, Jinling Hospital, Nanjing University School of Medicine, Nanjing 210002, China; ^5^ Pennsylvania State University, University Park, Pennsylvania, 16802, USA

**Keywords:** β-arrestin2, CRCLM, CXCR4, ubiquitination, USP33

## Abstract

Patients with liver metastases of colorectal cancer (CRCLM) have a poorer prognosis compared to colorectal cancer (CRC) patients in local stage. Evaluating the recurrence and overall survival of advanced patients is critical in improving disease treatment and clinical outcome. Here we investigated the expression pattern of USP33, a deubiquitinating enzyme, in both primary CRC tissues and liver metastases tissues. Univariate and multivariate analyses identified that low expression of USP33 in CRCLM tissues indicated high recurrence risk and poor overall prognosis. Overexpression of USP33 can significantly inhibit cell proliferation, migration, and invasion. On the other hand, USP33 knock-down promoted cell proliferation and invasion under SDF-1 stimulation; whereas dynasore (an internalization inhibitor) pretreatment in USP33 silencing cells showed a distinct antipromoting effect, revealing the participation of CXCR4 internalization in regulating tumor progress. Further results verified that USP33 can deubiquitinate β-arrestin2, subsequently block the internalization of SDF-1-stimulated CXCR4, and disrupt β-arrestin-dependent ERK activation. The existence and functions of β-arrestin-dependent signaling have been previously determined in several Gs-coupled receptors, such as β2-adrenergic receptor and angiotensin receptor subtype 1a; however, little is known about this in Gi-coupled receptors. Our study not only established USP33 as a novel prognosis biomarker in advanced CRCLM patients, but also highlighted the significance of β-arrestin-dependent ERK signaling in cancer development.

## INTRODUCTION

Colorectal cancer (CRC) is one of the most prevalent cancers and the third leading cause of cancer death in the world [1]. Although the 5-year overall survival (OS) rate of CRC is about 65% [2], it shows significant stage-dependence. For the patients who were diagnosed at a localized stage (less than 40% of all cases), the 5-year OS can be more than 90%. However, about 20% of patients have distant metastasis at the time of diagnosis, with the 5-year OS less than 15% [1]. The majority of CRC metastases localize to the liver, and surgical resection is the most effective therapy for liver metastases of CRC (CRCLM). For those patients, the biological features of both CRC and CRCLM are important in evaluating the clinical outcome. Moreover, even for the patients who underwent resection of CRC and CRCLM, 50–75% of cases may develop recurrence, especially intrahepatic recurrence [3, 4]. Therefore, determining the prognostic factors for patients with CRCLM is particularly important to improve the clinical outcome of these advanced cases.

Ubiquitination is one of the protein post-translational modifications which plays an important role in the regulation of cell proliferation, differentiation, and apoptosis [5]. We have previously demonstrated the role of ubiquitin ligase FBXW7 in regulating the epithelial-mesenchymal transition (EMT) in cancer metastasis [6]. On the other hand, the deubiquitination process, counteracting the ubiquitination by detaching ubiquitin and stabilizing the target substrate, is also drawing more and more attention. Ubiquitin-specific proteases (USPs) make up the largest family of deubiquitinating enzymes (DUBs), and several USPs, such as USP7 [7] and USP33 [8, 9], have been reported to play roles in regulating cancer development. USP33 can regulate centrosome amplication [10], recycling of β-adrenergic receptor [11, 12], and mediate Slit-Robo signaling in carcinoma [8, 13]. However, the protein expression of USP33 in CRC and CRCLM in advanced stage patients and their relationship with OS and disease-free survival (DFS) have not yet been investigated.

In the current study, we explored the expression pattern of USP33 in CRC and CRCLM tissues from patient who underwent resection of primary colorectal tumor as well as liver metastases. Our results showed that the expression level of USP33 in CRCLM, other than that in primary CRC, can act as an independent prognostic factor for the OS and DFS. In addition, *in vitro* study also demonstrated the role of USP33 in down-regulating cell proliferation, migration, and invasion.

Stromal cell-derived factor-1 (SDF-1) and its receptor, C-X-C chemokine receptor type 4 (CXCR4), are key regulators for cancer metastasis [14, 15]. Therefore, we sought to determine whether USP33 has cross-talk with SDF-1/CXCR4 signaling. Interestingly, our results demonstrated that USP33 knock-down can significantly increase the “late” ERK activation induced by SDF- 1. The prolonged duration of ERK activation has been reported to be β-arrestin-dependent in Gs-coupled GPCR signaling, such as β2-adrenergic receptor in HEK293 cells [16]. Here we demonstrated that this “late” ERK signaling by SDF-1/CXCR4 was also internalization and β-arrestin2-dependent, proving the existence of β-arrestin-dependent ERK signaling from Gi-coupled GPCR. Further experiments revealed that USP33 silencing, which increased the ubiquitination level of β-arrestin2, can lead to non-degradative effects towards this scaffold protein, but augment the endocytosis of ligand-binding CXCR4. Our results, which provide evidence for β-arrestin-dependent ERK signaling in cancer development and metastasis, emphasize the importance and broad prospects of biased-ligand development for GPCRs in cancer therapy.

## RESULTS

### Patient characteristics

The clinicopathological features of 139 CRCLM patients were listed in Table 1. The median patients' age was 61 years (range 31–81), and 87 patients (62.6%) were male. Eighty-six patients (61.9%) showed primary colon cancer, and the other 53 patients (38.1%) had primary rectum cancer. As for the primary CRC tumor differentiation, 11.5% showed poor differentiation, 73.4% showed moderate differentiation, and the other 15.1% showed well differentiation. Only 24 cases (17.3%) staged with T1–T2 according to the tumor filtration, while the other 115 cases (82.7%) were staged as T3-T4 at the time of primary CRC tumor resection.

**Table 1:** Correlation between USP33 expression and clinicopathologic characteristics of CRCLM patients

Variable	*n*	USP33 expression in primary tumor		*P*	USP33 expression in CRCLM		*P*
		Low (*N* = 86)	High (*N* = 53)		Low (*N* = 54)	High (*N* = 85)	
Gender				0.672			0.314
Female	52	31	21		23	29	
Male	87	55	32		31	56	
Age (year)				0.700			0.796
< 50	42	27	15		17	25	
≥ 50	97	59	38		37	60	
Primary tumor location				0.664			0.388
Colon	86	52	34		31	55	
Rectum	53	34	19		23	30	
Primary tumor differentiation				0.771			0.256
Poor	16	11	5		9	7	
Moderate	102	63	39		36	66	
Well	21	12	9		9	12	
Primary tumor staging				0.002*			0.001*
T1–T2	24	8	16		2	22	
T3–T4	115	78	37		52	63	
Preoperative CEA level				0.754			0.222
< 100 ng/mL	81	51	30		30	56	
≥ 100 ng/mL	58	35	23		24	29	
Distribution of CRCLM				0.808			0.682
Unilobar	98	60	38		37	61	
Bilobar	41	26	15		17	24	
No. of CRCLM				0.002*			0.269
< 3	90	47	43		38	52	
≥ 3	49	39	10		16	33	
Largest diameter of CRCLM				0.647			0.678
< 5 cm	69	44	25		28	41	
≥ 5 cm	70	42	28		26	44	
Differentiation of CRCLM				0.188			0.713
Poor	6	4	2		3	3	
Moderate	120	77	43		47	73	
Well	13	5	8		4	9	
Resection margin of CRCLM				0.256			0.888
R1	15	7	8		5	10	
R0, 1–10 mm	87	58	29		34	53	
R0, > 10 mm	37	21	16		15	22	
LN metastasis				0.001*			< 0.001*
Negative	36	14	22		5	31	
Positive	103	72	31		49	54	
Extrahepatic metastasis				0.701			0.087
Negative	120	75	45		50	70	
Positive	19	11	8		4	15	

Abbreviations: CRCLM: liver metastases of colorectal cancer; CEA: carcinoembryonic antigen; T1: stage I; T2: stage II; T3: stage III; T4: stage IV.

The characteristics about liver metastases (CRCLM) at the time of hepatectomy were also retrieved. Nighty-eight cases (70.5%) had unilobar metastases, and other 41 patients (29.5%) had bilobar metastases. For 90 patients (64.7%) among the cohort, the number of CRCLM was less than 3, and the other 49 patients (35.3%) had more than 3 metastases. The largest diameter of CRCLM was 4.3 ± 2.3 cm (range 0.6–8.3 cm). Consistent with the differentiation of primary CRC tumor, most of the patients (86.3%) showed moderate differentiation of CRCLM. For the surgical procedure towards CRCLM, 89.2% patients underwent the R0 resection, and the other 10.8% patients received R1 resection. Most patients (74.1%) suffered with positive lymph node (LN) metastasis, while only 13.7% patients showed positive extrahepatic metastasis.

### Expression of USP33 and its association with clinicopathological characteristics

**Figure 1:**
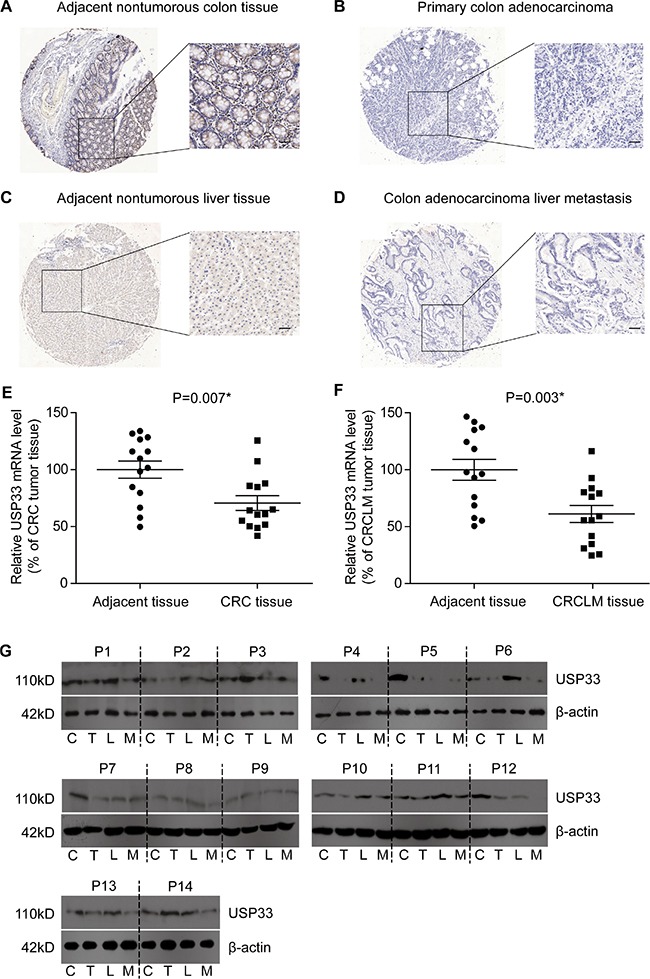
The expression patterns of USP33 in CRC and CRCLM tissues Representative immunostaining of USP33 expression in adjacent nontumorous colon tissue (**A**, high expression), primary CRC tissue (**B**, low expression), adjacent nontumorous liver tissue (**C**, high expression), and CRCLM tissue (**D**, low expression) by IHC analysis. Fresh-frozen specimens from 14 patients showed that mRNA level of USP33 in CRC tissue was lower than that of adjacent nontumorous colon tissue (**E**). Similarly, the USP33 mRNA level in CRCLM tissue was significantly lower compared to adjacent liver tissue (**F**). The protein level of USP33 in fresh-frozen tissues was also assessed by Western Blot (**G**). (scale bar: 100 μM)

Expression and subcellular localization of USP33 protein were determined by IHC towards the CRC tissues, CRCLM tissues, adjacent non-tumorous colon tissues, and adjacent non-tumorous liver tissues. USP33 was mainly expressed in the cytoplasm and was significantly down-regulated in both the CRC and CRCLM tissues, compared with adjacent non-tumorous tissues (Figure 1A–1D). In addition, we evaluated the expression of USP33 on both RNA level (Figure 1E, 1F) and protein level (Figure 1G) from another 14 paired fresh-frozen tissues, which showed that USP33 expression in tumor tissues was lower than that in adjacent non-tumorous tissues. The down-regulation of USP33 in clinical specimens indicated that the USP33 may act as a tumor suppressor in the CRC development.

To further investigate the correlation between USP33 expression and patients' characteristics, we grouped the patients into USP33 low-expression and USP33 high-expression according to the immunoreactivity. Low-expression of USP33 in CRC and CRCLM tissues was observed in 86 patients (61.9%) and 54 patients (38.8%), respectively. USP33 low-expression in CRC and CRCLM were both correlated with advanced tumor stage and positive LN metastasis (Table 1). Importantly, the USP33 expression in primary CRC, but not CRCLM, is associated with the number of liver metastases (Table 1). This association can be explained by the fact that the primary tumor biological features, other than that of metastases, may be more dominant in controlling the tumor metastasis process.

### USP33 can act as a novel prognostic factor for OS and DFS of CRCLM patients

The median OS time for all patients was 39.5 months (range 3–167 months), and the median DFS time was 33 months (range 4–167 months). The 1-year, 3-year, and 5-year OS of all patients were 79.1%, 55.2%, and 39.8%, respectively. The 1-year, 3-year, and 5-year DFS were 69.8%, 47.3%, and 31.1%, respectively.

**Figure 2:**
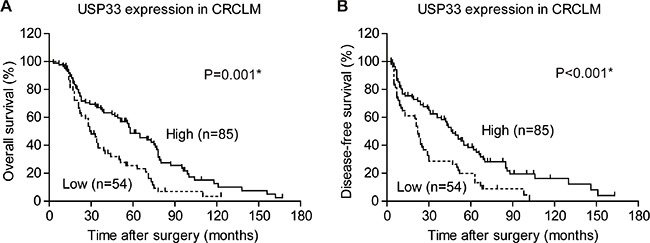
Kaplan-Meier survival curve according to USP33 expression in CRCLM tissues (**A**) The 5-year overall survival in the patients with high USP33 expression and low USP33 expression were 48.6% and 25.6%, respectively (*P* = 0.001). (**B**) The 3-year disease free survival of patients in the high USP33 expression group and low USP33 expression group were 59.8% and 28.7%, respectively (*P* < 0.001).

Using Kaplan-Meier survival analysis (Table 2), we identified that bilobar (*P* = 0.001), multi-number (*P* = 0.010), large diameter (*P* = 0.009), poor differentiation (*P* = 0.018), and positive resection margin (*P* < 0.001) of CRCLM were all correlated with poor OS. Neither the location, differentiation, nor filtration stage of primary CRC showed prognostic significance. Moreover, the low-expression of USP33 in either CRC or CRCLM was significantly correlated with poor clinical outcome (Figure 2A). Other prognostic factors for OS include LN metastasis and extrahepatic metastasis. Neither the location, differentiation, or filtration stage of primary CRC showed prognostic significance.

**Table 2:** The overall survival was analyzed with Kaplan-Meier univariate analysis

Variable	Cases (*n*)	5-year OS rate (%)	OS time (months) (mean ± s.d)	*P* value
Gender				0.539
Female	52	46.29	58.57 ± 6.04	
Male	87	35.89	54.00 ± 4.66	
Age (year)				0.378
< 50	42	52.42	61.31 ± 7.26	
≥ 50	97	34.83	53.87 ± 4.43	
Primary tumor location				0.107
Colon	86	43.65	60.88 ± 5.01	
Rectum	53	33.55	47.93 ± 5.36	
Primary tumor differentiation				0.704
Poor	16	40.10	47.78 ± 6.30	
Moderate	102	38.26	55.40 ± 4.47	
Well	21	46.56	61.93 ± 9.82	
Primary tumor staging				0.067
T1–T2	24	52.65	73.59 ± 12.12	
T3–T4	115	37.07	53.45 ± 3.70	
Preoperative CEA level				0.604
< 100 ng/mL	81	43.99	57.31 ± 4.41	
≥ 100 ng/mL	58	33.82	56.79 ± 7.37	
Distribution of CRCLM				0.001*
Unilobar	98	47.32	62.89 ± 4.67	
Bilobar	41	21.32	39.72 ± 5.68	
No. of CRCLM				0.010*
< 3	90	47.11	62.43 ± 4.60	
≥ 3	49	25.85	42.18 ± 5.25	
Largest diameter of CRCLM				0.009*
< 5 cm	69	51.11	65.59 ± 5.44	
≥ 5 cm	70	21.83	47.23 ± 5.20	
Differentiation of CRCLM				0.018*
Poor	6	16.67	33.67 ± 11.94	
Moderate	120	36.83	52.80 ± 3.62	
Well	13	69.23	88.04 ± 16.38	
Resection margin of CRCLM				< 0.001*
R1	15	6.67	17.68 ± 2.38	
R0, 1 - 10 mm	87	41.61	55.39 ± 3.73	
R0, > 10 mm	37	49.02	66.28 ± 8.17	
LN metastasis				< 0.001*
Negative	36	58.39	83.95 ± 8.17	
Positive	103	33.78	45.49 ± 3.50	
Extrahepatic metastasis				< 0.001*
Negative	120	43.61	60.17 ± 4.07	
Positive	19	15.79	30.26 ± 8.74	
USP33 expression in primary tumor				0.044*
Low	86	31.49	51.05 ± 5.12	
High	53	44.81	59.63 ± 4.31	
USP33 expression in CRCLM				0.001*
Low	54	25.58	40.94 ± 4.09	
High	85	48.60	65.01 ± 5.19	

Abbreviations: OS: overall survival; CRCLM: liver metastases of colorectal cancer; CEA: carcinoembryonic antigen; T1: stage I; T2: stage II; T3: stage III; T4: stage IV.

Probable risk factors of recurrence were also evaluated by univariate analysis (Table 3). The distribution (*P* = 0.045), number (*P* < 0.001), largest diameter (*P* = 0.020), differentiation (*P* = 0.047), and resection margin (*P* < 0.001) of CRCLM were all predictive aspects for DFS. Besides, the primary CRC tumor stage (*P* = 0.019), LN metastasis (*P* < 0.001), and USP33 expression in CRCLM (*P* < 0.001, Figure 2B) were also associated with the disease relapse.

**Table 3:** The disease-free survival for different clinicopathological factors by univariate analysis

Variable	Cases (n)	3-year DFS rate (%)	DFS time (months) (mean ± s.d)	*p* value
Gender				0.507
Female	52	47.28	42.85 ± 5.29	
Male	87	48.44	49.76 ± 5.58	
Age (year)				0.277
< 50	42	51.88	58.56 ± 8.98	
≥ 50	97	46.23	43.89 ± 4.37	
Primary tumor location				0.141
Colon	86	52.69	51.65 ± 5.50	
Rectum	53	39.97	38.86 ± 5.23	
Primary tumor differentiation				0.507
Poor	16	60.00	35.46 ± 6.00	
Moderate	102	45.19	47.44 ± 4.77	
Well	21	52.38	52.17 ± 11.46	
Primary tumor staging				0.019*
T1 - T2	24	54.11	70.44 ± 14.34	
T3 - T4	115	46.84	42.19 ± 3.68	
Preoperative CEA level				0.905
< 100 ng/mL	81	49.62	47.34 ± 4.91	
≥ 100 ng/mL	58	43.99	48.07 ± 7.20	
Distribution of CRCLM				0.045*
Unilobar	98	53.58	51.53 ± 4.86	
Bilobar	41	32.53	36.06 ± 6.93	
No. of CRCLM				< 0.001*
< 3	90	56.79	56.72 ± 5.47	
≥ 3	49	31.25	29.19 ± 4.12	
Largest diameter of CRCLM				0.020*
< 5 cm	69	56.31	57.54 ± 6.49	
≥ 5 cm	70	37.65	38.00 ± 4.89	
Differentiation of CRCLM				0.047*
Poor	6	25.00	30.42 ± 11.30	
Moderate	120	45.81	42.83 ± 3.67	
Well	13	61.54	77.12 ± 17.83	
Resection margin of CRCLM				< 0.001*
R1	15	0	9.05 ± 1.58	
R0, 1–10 mm	87	52.26	43.82 ± 3.68	
R0, > 10 mm	37	49.27	60.13 ± 9.03	
LN metastasis				< 0.001*
Negative	36	79.76	81.63 ± 9.29	
Positive	103	35.62	34.36 ± 3.43	
Extrahepatic metastasis				0.211
Negative	120	48.03	45.40 ± 4.28	
Positive	19	51.40	29.74 ± 14.93	
USP33 expression in primary tumor				0.111
Low	86	46.26	44.02 ± 5.32	
High	53	49.01	47.34 ± 4.58	
USP33 expression in CRCLM				< 0.001*
Low	54	28.67	30.88 ± 4.05	
High	85	59.81	58.23 ± 5.81	

Abbreviations: DFS: disease-free survival; CRCLM: liver metastases of colorectal cancer; CEA: carcinoembryonic antigen; T1: stage I; T2: stage II; T3: stage III; T4: stage IV.

Moreover, multivariate analysis verified that the low-expression of USP33 in CRCLM, but not in CRC, can serve as an independent prognostic factor for both OS and DFS (Table 4). This implied that the status of liver metastases may be more valuable than that of primary tumor during the prognosis evaluation for CRCLM patients. Other independent prognostic factors were listed in Table 4.

**Table 4:** Multivariate Cox-regression analysis of overall survival and disease-free survival

OS			
Variable	HR	95% CI	*P* value
Distribution of CRCLM	1.988	1.296 − 3.049	0.002*
No. of CRCLM	1.923	1.265 − 2.923	0.002*
Largest diameter of CRCLM	1.550	1.049 − 2.291	0.028*
Differentiation of CRCLM	0.836	0.489 − 1.431	0.514
Resection margin of CRCLM	0.592	0.397 − 0.882	0.010*
LN metastasis	1.658	1.005 − 2.736	0.048*
Extrahepatic metastasis	2.238	1.282 − 3.904	0.005*
USP33 expression in primary tumor	1.355	0.887 − 2.068	0.160
USP33 expression in CRCLM	1.653	1.113 − 2.453	0.013*
DFS			
**Variable**	**HR**	**95% CI**	***P* value**
Primary tumor staging	1.430	0.770 − 2.655	0.257
Distribution of CRCLM	1.574	1.019 − 2.432	0.041*
No. of CRCLM	2.128	1.409 − 3.216	< 0.001*
Largest diameter of CRCLM	1.513	1.020 − 2.242	0.039*
Differentiation of CRCLM	0.814	0.486 − 1.365	0.436
Resection margin of CRCLM	0.638	0.425 − 0.959	0.031*
LN metastasis	2.425	1.443 − 4.075	0.001*
USP33 expression in CRCLM	1.596	1.055 − 2.412	0.027*

Abbreviations: OS: overall survival; DFS: disease-free survival; HR: hazard ratio; CI: confidence interval; CRCLM: liver metastases of colorectal cancer.

### USP33 inhibits cell proliferation, migration, and invasion

Since the clinical data showed that USP33 plays important roles in the recurrence and survival of CRC patients, we carried out *in vitro* experiments using CRC cell lines to further explore the underlying mechanism of its functions.

**Figure 3:**
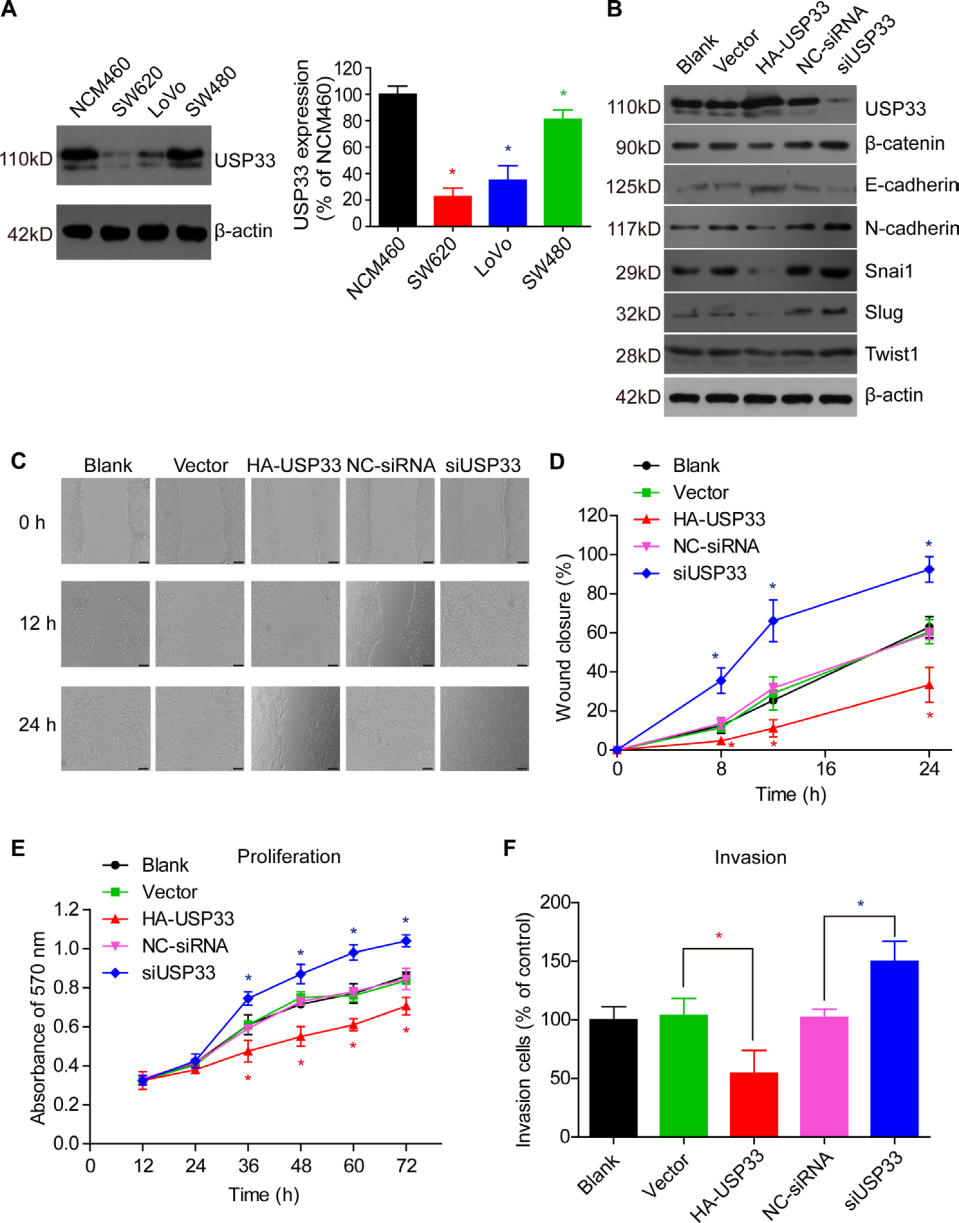
USP33 inhibits cell proliferation, migration, and invasion (**A**) The expression of USP33 in different cell lines was detected using Western Bolt. The NCM460 normal colonic epithelial cells showed higher USP33 expression compared to colon cancer cell lines. (**B**) USP33 expression in LoVo cells was determined 48 h after overexpression or siRNA knock-down. USP33 overexpression significantly down-regulated the protein levels of β-catenin, snai1, slug, twist1, and N-cadherin, while increasing E-cadherin expression. Silencing of USP33 showed reverse effects towards the EMT protein expression pattern, compared to USP33 overexpression. (**C**) The effect of USP33 in regulating cell migration was measured in LoVo cells by a wound healing assay under the stimulation of SDF-1 (100 ng/mL). (**D**) Migration rate was evaluated by the percentage of wound closure [(initial width - terminal width)/ initial width*100%] according to the images in (C). The results showed that USP33 knock-down can significantly promote cell migration, while USP33 overexpression exhibited a down-regulation effect. (**E**) Cell proliferation was detected by a MTT-assay, showing how USP33 expression was negatively correlated with cell proliferation. (**F**) Silencing of USP33 can increase the cell invasive capacity under SDF-1 stimulation, whereas USP33 overexpression demonstrated the opposite regulation. The data was repeated three times and presented as mean ± S.D. (*P < 0.05, compared with control, calculated by *t-test*).

We measured the protein expression levels of USP33 in normal colonic epithelial cells (NCM460) and various CRC cell lines (SW480, SW620, LoVo). Western Blot results showed that NCM460 has the highest USP33 expression, which is consistent with the IHC results; LoVo cells showed moderate USP33 expression among all the cell lines (Figure 3A). Taking into consideration that LoVo cells were derived from the metastatic site of CRC, we therefore chose LoVo cells to perform the overexpression and knock-down assays. The transfection efficiency as well as the level of EMT proteins were examined by Western Blot. We found that USP33 overexpression can significantly down-regulate the protein level of β-catenin, snai1, slug, twist1, and N-cadherin, while increasing the E-cadherin expression (Figure 3B). On the other hand, silencing USP33 exhibited reciprocal regulation effects towards the EMT proteins.

Moreover, overexpression of USP33 resulted in a significant decrease in the proliferative and invasive capacity of CRC cells, as determined by MTT assay, wound healing assay, and transwell invasion assay (Figure 3C–3E). In contrast, USP33 knock-down showed positive regulation towards the cell viability and invasion.

### USP33 regulates internalization and recycling of CXCR4

It has been reported that USP33 can promote the internalization of β2-adrenergic receptor and vasopressin receptor 2 [11, 12]. One of the most important GPCRs in cancer migration and invasion is CXCR4, however, the correlation between CXCR4 and USP33 hasn't been investigated. We therefore tried to figure out whether there has any cross-talk between USP33 and CXCR4 in CRC development.

We found that the expression of CXCR4 in tumor tissues was higher than that in adjacent non-tumorous tissues (Supplementary Figure S1A–S1D). Interestingly, the average IHC score of CXCR4 in CRCLM tissues was lower than that in primary CRC tissues (*P* = 0.03, data not shown), indicating the expression of CXCR4 may be down-regulated during migration process. One generalities of GPCR is the receptor internalization under the ligand stimulation, and SDF-1 is the primary stimuli for CXCR4. We assumed that the stimulation of SDF-1 can promote tumor cell metastasis, and long-time stimulation can contribute to the down-regulation of CXCR4 in CRCLM.

**Figure 4:**
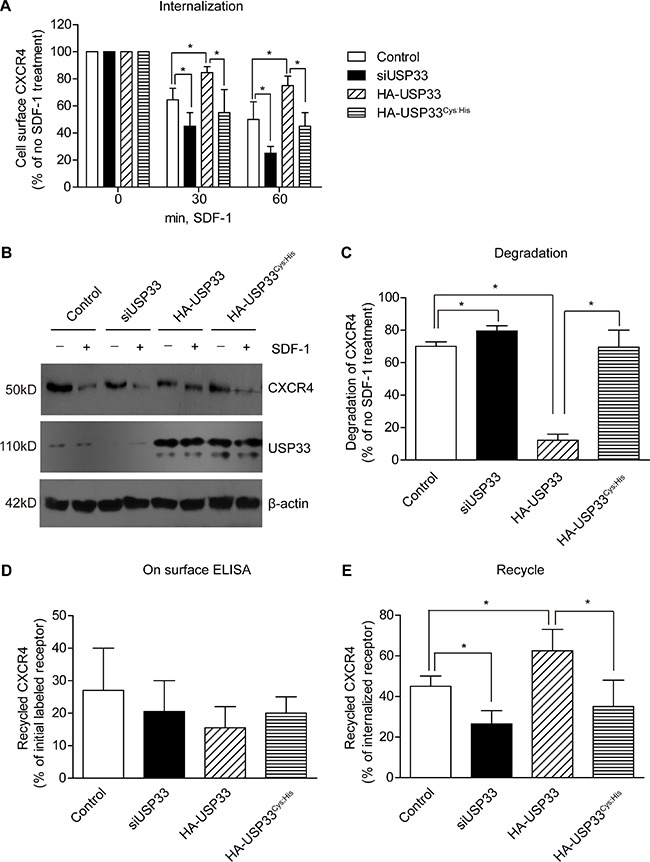
Silencing USP33 promotes the agonist-induced endocytosis and degradation of CXCR4 (**A**) LoVo cells transfected with HA-USP33 or USP33-siRNA were stimulated with 100 ng/mL SDF-1 for the indicated times, and receptor internalization was quantified using on surface ELISA. CXCR4 membrane expression level was up-regulated with USP33 overexpression, while the expression level was down-regulated with USP33 knock-down. (**B**) USP33 prevents degradation of CXCR4. The amount of receptor from total cell lysates was determined by Western Blot after stimulation with 100 ng/mL SDF-1 for 1 h, and the results showed that CXCR4 overexpression can significantly inhibit degradation of internalized receptors. (**C**) The results from (B) was semi-quantified with Image J software. (**D, E**) The amount of recycled receptors was detected by ELISA as described in *Patients and Methods*. Even the total amount of recycled labeled receptors showed no difference (D), the exact recycled fraction versus internalized receptors provided remarkable significance [**E**, results were presented as (the amount of surface receptors in Figure D)/ (the amount of internalized receptors by Figure A)]. All experiments were performed in triplicate, and the data (mean ± S.D., *P < 0.05, calculated by *t-test*) came from three independent experiments.

The LoVo cells showed detectable endogenous CXCR4 (Supplementary Figure S1E, S1F), and the role of USP33 in regulating CXCR4 expression was further investigated. We demonstrated that USP33 overexpression can attenuate CXCR4 internalization, while USP33 knock-down showed the opposite effect (Figure 4A). To evaluate whether the DUB enzyme activity of USP33 was required for inhibiting CXCR4 internalization, we generated the mutant construct USP33^Cys:His^, which has been verified as inactive USP33 mutant [11]. In cells transfected with USP33^Cys:His^, the internalization extent was similar with control group, meaning the USP33 DUB activity is fundamental in preventing receptor internalization.

On the other hand, we tested the level of total receptors after SDF-1 stimulation and demonstrated that USP33 knock-down can increase the degradation of CXCR4, while USP33 overexpression can inhibit its degradation (Figure 4B, 4C). Furthermore, we performed on surface ELISA to evaluate receptor internalization as well as the recycle ratio of internalized receptors, which interestingly demonstrated that USP33 knock-down can not only promote internalization (Figure 4D), but also impair the recycling of CXCR4 (Figure 4E).

### USP33 leads to de-ubiquitination of β-arrestin2 with non-degradative effects

The GPCR desensitization and internalization process has been reviewed elsewhere [17]. Briefly, activated GPCRs triggered the G-protein-mediated signaling, and are subsequently phosphorylated, which leads to β-arrestin recruitment to the ligand-bound, phosphorylated receptor to un-couple the G protein mediated signaling (desensitization). β-arrestin can serve as a scaffold protein and recruit a number of other proteins such as AP-2, clathrin, and myosin, thus promoting the endocytosis of GPCRs (internalization). As for CXCR4, the primary β-arrestin isoform in mediating receptor internalization is β-arrestin2 [18, 19].

**Figure 5:**
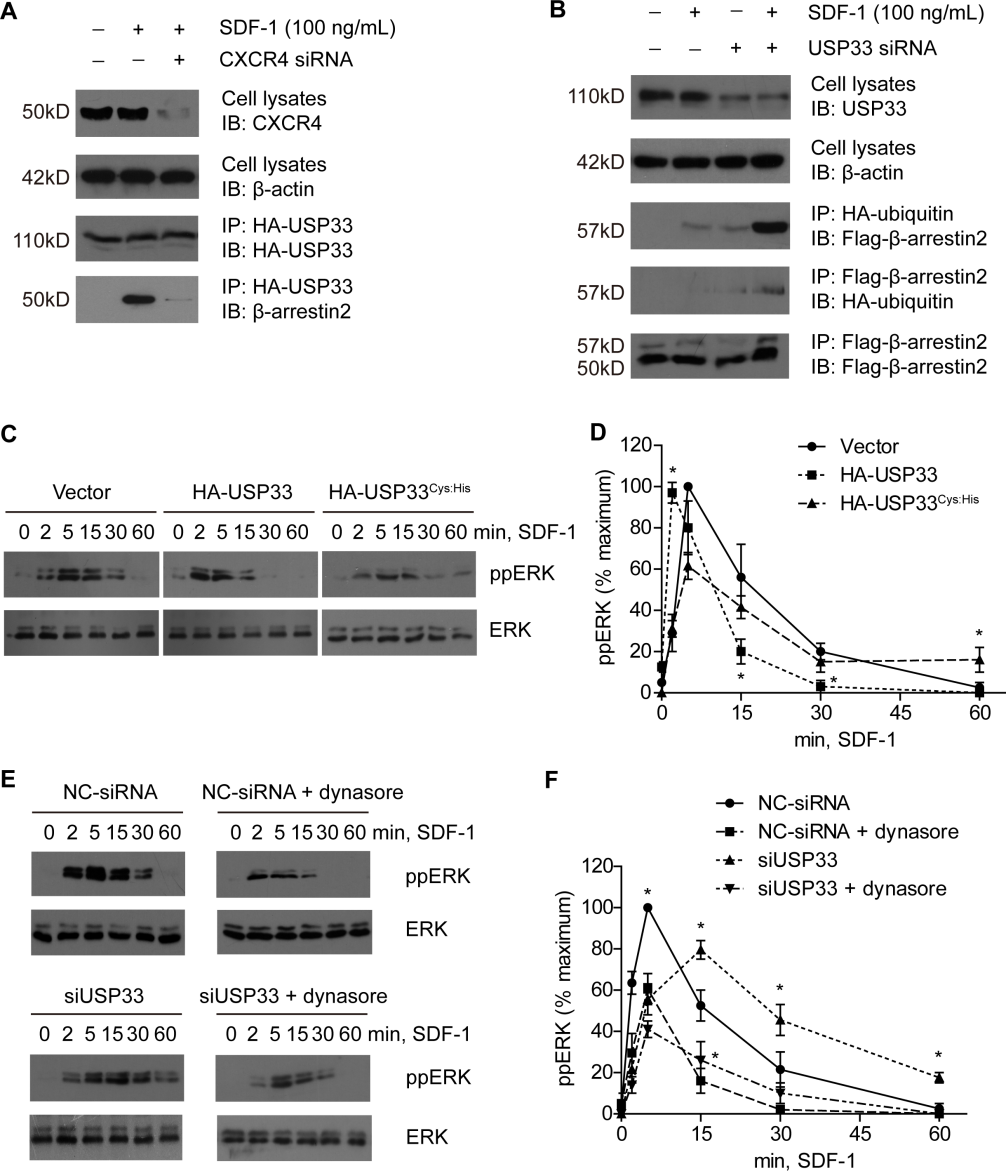
USP33 mediates the duration of SDF-1-stimulated EKR signaling through deubiquitinating β-arrestin2 (**A**) USP33 can interact with β-arrestin2 in an agonist-receptor dependent manner. HA-tagged USP33 expressed in LoVo cells (with or without CXCR4 knock-down) was immunoprecipated with HA affinity beads after stimulation with 100 ng/mL SDF-1 for 15 min. Transfection efficiency of CXCR4-siRNA, as well as the presence of USP33 and β-arrestin2 in the IP, were determined by Western Blot. SDF-1 stimulation can induce the interaction between USP33 and β-arrestin2, while CXCR4-siRNA can diminish, if not abolish, this interaction. (**B**) USP33 regulates the ubiquitination status of β-arrestin2. LoVo cells were co-transfected with Flag-β-arrestin2 and HA-ubiquitin plasmids, with or without silencing USP33, as described in *Patients and Methods*. After stimulation with 100 ng/mL SDF-1 for 1 h, cells were harvested and immunoprecipitation was performed with HA affinity beads and Flag affinity beads, respectively. Under stimulation with SDF-1, Flag-β-arrestin2 was detected from HA-IP, indicating β-arrestin2 was ubiquitinated after SDF-1/CXCR4 stimulation. Moreover, USP33 knock-down significantly increased the immunoreactivity of Flag-β-arrestin2 in the HA-IP, meaning that USP33 can deubiquitinate β-arrestin2. Similar results were found from Flag-IP, being that USP33 knock-down plus SDF-1 stimulation increased interaction between Flag-β-arrestin2 and HA-ubiquitin. Also the molecular weight of β-arrestin2 was increased, demonstrating its ubiquitinated status. (**C**) Activation of ERK was determined by Western Blot analysis. After transfection for 48 h, LoVo cells were starved overnight and stimulated with 100 ng/mL SDF-1 for the indicated times. The activated ERK in whole cell lysates was detected with phosphor-ERK (p-p42/44) antibody. Total ERK was also detected as normalization. (**D**) Time course of ERK activation was plotted, showing the “left shift” effect of USP33 overexpression. Transfection with the inactive USP33 mutant, USP33^Cys:His^, can increase the “late” ERK signaling compared to wild type USP33. (**E**) USP33 siRNA significantly extended the duration of ERK signaling, indicating a “right shift” effect. However, pretreatment with dynasore, an internalization inhibitor, can attenuate the “late” ERK signaling in both control cells and USP33-silencing cells, meaning the “late” ERK signaling may be dependent on receptor internalization. (**F**) Relative intensity of phosphorylated ERK from (**E**) was semi-quantified using Image J software and normalized by total ERK1/2. All results are presented as mean ± S.D. of triplicate samples and are representative of three independent experiments (*P < 0.05, compared with control, calculated by *t-test*).

Given that the enzymatic activity of USP33 is important in regulating CXCR4 internalization, we were interested in whether USP33 can deubiquitinate β-arrestin2 and subsequently regulate CXCR4 endocytosis. The immunoprecipitation assay showed that USP33 can interact with β-arrestin2 in an agonist-dependent manner (Figure 5A). Moreover, USP33 knock-down can significantly increase the ubiquitination level of β-arrestin2 under SDF-1 stimulation (Figure 5B). However, the protein level of β-arrestin2 was not significantly affected by USP33, suggesting that the β-arrestin2 ubiquitination status may influence its activity instead of proteasome-degradation process, which is consistent with others' results [20–22].

In addition, we investigated the expression of β-arrestin2 in clinical specimens by IHC. Although β-arrestin2 was up-regulated in both primary CRC tissues and liver metastases (Supplementary Figure S2A–S2D), there was no significant correlation between the USP33 and β-arrestin2 expression levels (Supplementary Figure S2E, S2F). The IHC results also indicated that USP33 may not directly regulate the expression or degradation of β-arrestin2.

### Effect of USP33 on the duration of ERK signaling

MAPK signaling is the predominant downstream pathway regulated by SDF-1/CXCR4 axis, therefore, we wondered if USP33 can influent MAPK signaling.

We observed that USP33 overexpression can shorten the duration of ERK signaling (Figure 5C) induced by SDF-1, as well as “left shift” the peak of ERK signaling. However, the ERK signaling was prolonged in cells transfected with USP33^Cys:His^, opposite the effect of wild type USP33 overexpression (Figure 5C, 5D). Moreover, overexpression of USP33^Cys:His^ can slightly promote cell proliferation and invasion (Supplementary Figure S3A, S3B), instead of the inhibiting effects caused by USP33 wild type. This opposite effect implicated that the USP33^Cys:His^ showed a competitive inhibition effect towards the endogenous USP33; in other words, the USP33 enzyme activity is critical in inhibiting ERK signaling and proliferation, which encouraged us to further investigate the underlying mechanisms in its cross-talk with SDF-1/CXCR4-ERK signaling.

We further performed the USP33 knock-down experiments to assess the changes of ERK signaling. As expected, the ERK signaling was prolonged and “right shift” in the cells transfected with USP33 siRNA (Figure 5E, left panels). However, the highest intensity of ppERK was lower than that in cells transfected with negative-control siRNA. Therefore, we conclude the influences of USP33-siRNA on ERK signaling as: 1) prolong the ERK signaling; 2) “right shift” the peak of ERK signaling; and 3) decrease the highest intensity of ERK signaling induced by SDF-1 (Figure 5F).

### USP33 regulates β-arrestin2-dependent ERK signaling induced by SDF-1/CXCR4

We have proved that USP33 knock-down can promote the internalization and degradation of CXCR4 (Figure 4). However, it's contradictive that SDF-1 mediated ERK singling was prolonged but not shortened by silencing USP33 (Figure 5D). It has been reported that during β-arrestin-dependent endocytosis, some kinds of GPCRs will trigger a second cascade intracellular signaling through β-arrestin (β-arrestin-dependent signaling) [23, 24]. The β-arrestin-dependent signaling has been extensively studied in the β2-adrenergic receptor, in which it was demonstrated that the receptor can generate another ERK signaling cascade (“late” ERK signaling, usually after 15 min stimulation by isoproterenol) [25]. Hence, it's likely that the prolonged ERK signaling in our results is also caused by β-arrestin.

Another important argument is that the inhibition of β-arrestin ubiquitination can impair agonist-stimulated receptor internalization and β-arrestin-dependent signaling in Gq-coupled receptors [21, 22, 26]. Therefore, we further tested whether the prolonged ERK signaling by USP33-siRNA was caused by internalized CXCR4. We found that dynasore, an efficient internalization inhibitor, can significantly inhibit the “late” ERK signaling (Figure 5E, 5F). In addition, pretreatment with dynasore can also attenuate the effects of USP33-siRNA in promoting cell proliferation and invasion (Supplementary Figure S3C, S3D), indicating that the USP33's role as a tumor suppressor is at least partially mediated by β-arrestin-dependent ERK signaling.

We also demonstrated that in the LoVo cells transfected with CXCR4, overexpression of β-arrestin2 can further promote cell proliferation, while co-overexpression of USP33 can attenuate the oncogenetic effects of CXCR4 (Supplementary Figure S4A, S4B). The distinct functions of β-arrestin2 and USP33 implicated the importance of these two molecules in CRC development.

## DISCUSSION

It has been recently reported that low USP33 expression in primary CRC tissue was correlated with poor prognosis [13]; however, we found that for advanced patients with CRCLM, the low USP33 expression in primary CRC tissue has no prognostic significance; instead, the expression of USP33 in liver metastases can serve as a predictive factor for both disease recurrence as well as overall survival. The difference between the two studies is not contradictive. For general CRC patients, the biological characteristics of primary CRC are the most important impact factors in predicting clinical outcome, but for advanced CRC patients who have already occurred the liver metastases, the status of CRCLM can be more valuable during both treatment and prognosis evaluation. On the other hand, due to the complicated process of tumor migration and liver specific microenvironments, the tumor cells in liver metastases can show distinct gene expression pattern and protein modifications.

Our results demonstrated that USP33 knock-down significantly prolonged SDF-1 induced ERK signaling in colorectal cancer cells by regulating the ubiquitination status of β-arrestin2. ERK downstream signaling plays important roles in mediating tumor proliferation and metastasis [27–29]; the intensity, duration, and subcellular localization of ERK activation are well regulated and provide different signaling specificity [30, 31]. We thus tested the function of USP33 in cell proliferation, migration, and invasion. Consistent with our clinical data, USP33 knock-down increased the carcinogenicity of cancer cells, such as up-regulating the EMT protein expression and promoting cell proliferation and invasion.

Taken together, USP33 expression was down-regulated in tumor tissues. and silencing USP33 can promote internalization and degradation of CXCR4. Therefore, it's reasonable if CXCR4 was subsequently low expressed in tumor tissues. However, we found that the CXCR4 level in tumor tissues was actually higher than that in adjacent non-tumorous tissues (Supplementary Figure S1A–S1D), and enhanced expression of CXCR4 was also reported to be associated with poor diagnosis in other malignant tumors [14, 15]. This contradictive phenomenon can be partly explained by the fact that CXCR4 expression level in liver metastases was lower than that in primary colorectal cancer tissues (Supplementary Figure S1B, S1D). One hypothesis is that during the SDF-1 induced migration of cancer cells, USP33 expression is down-regulated in certain microenvironments, which enhanced cell migration capacity as we discussed above. On the other hand, the consistent stimulation of SDF-1 and down-regulation of USP33 gradually promote the degradation of CXCR4, which contribute to the lower CXCR4 level in liver metastases than primary tumor tissues. According to this hypothesis, only the primary CRC cells that possess higher CXCR4 level can bear the consistent stimulation of SDF-1, indicating higher possibility to occur distant metastasis. To test this explanation, we compared the effects of USP33 knock-down in LoVo cells with or without CXCR4 overexpression. As expected, overexpression of CXCR4 can up-regulate cells' carcinogenicity in response to USP33-siRNA (Supplementary Figure S4C, S4D).

Accordingly, USP33 may indirectly regulate the degradation and recycling of CXCR4 through deubiquitinating β-arrestin2, therefore promote tumor cell metastasis, although a more detailed mechanism remains to be determined.

## MATERIALS AND METHODS

### Patients and specimens

This multi-center retrospective study was approved by the Ethics Committee of Shandong University, and written informed consent was obtained from all patients. We collected 139 CRC patients with synchronous liver metastases or early metachronous liver metastases (defined as liver metastases occurring within 12 months of the primary CRC diagnosis), all of whom underwent surgical resection of primary CRC and CRCLM from January 2000 to January 2013 at the Qilu Hospital, Qianfoshan Hospital, Shanxian Central Hospital or Yuncheng People's Hospital. Clinical information retrieved include gender, age, primary tumor location, primary tumor differentiation, primary tumor stage, preoperative CEA level, lymph node metastasis, extrahepatic metastasis; as well as the distribution, number, largest diameter, differentiation and resection margin of liver metastases.

Paraffin-embedded specimens of tumor tissues and adjacent non-tumorous tissues were obtained from the Department of Pathology. Representative 1.5-mm tissue cores from each specimen were used to conduct a tissue microarray (TMA) analysis as described previously [38] using the Quick-Ray Manual Tissue Microarrayer (UNITMA, Korea) [39]. TMA samples were then cut into 4-μm sections slides for the later immunohistochemical staining. Another 14 paired fresh-frozen tissues from patients with CRCLM were collected from 2014 to 2016, and stored in liquid-nitrogen until use.

### Reagents

The following antibodies were used in this study: anti-USP33, anti-slug, and anti-twist1 antibodies were purchased from ProteinTech Group (Chicago, IL, USA); anti-CXCR4 antibody was purchased from BD Bioscience (Franklin Lakes, NJ, USA); anti-HA and anti-Flag M1 antibodies were from Sigma-Aldrich (St. Louis, MO, United States); anti-ppERK, anti-ERK, and anti-β-arrestin2 antibodies were from Cell Signaling Technology (Boston, MA, USA); anti-E-cadherin, anti-N-cadherin, anti-snai1, and anti-β-actin antibodies were purchased from Santa Cruz (Dallas, TX, USA).

*N*-ethylmaleimide (NEM, deubiquitinase inhibitor), SDF-1 (SDF-1α, CXCR4 agonist), AMD3100 octahydrochloride hydrate (1,1′-[1,4-Phenylenebis(methylene)]bis-1,4,8,11-tetraazacyclotetradecane octahydrochloride, CXCR4 antagonist), dynasore hydrate (3-Hydroxy-naphthalene-2-carboxylic acid (3,4-dihydroxy-benzylidene)-hydrazide hydrate, dynamin inhibitor), HA affinity beads, and Flag affinity beads were all purchased from Sigma-Aldrich.

### Immunohistochemistry (IHC)

The IHC analysis of TMA specimens was performed as previously described [40] with the primary antibodies. Briefly, TMA slides sequentially underwent deparaffinization, rehydration, blockage, antigen retrieval, incubation with antibody, DAB staining, and hematoxylin counter-staining. The USP33 staining results were assessed and scored by two independent pathologists. The score for IHC staining intensity was classified into four categories: 0 (negative), 1 (weakly positive), 2 (moderately positive), and 3 (strongly positive). The score for the percentage of positive staining cells was also scaled as 0 (0–10%), 1 (11–25%), 2 for (26–75%), and 3 (76–100%). The overall quantification of staining score was generated by multiplying the average intensity and percentage score from five random selected fields, ranging 0–9. Finally, we grouped the USP33 into low (score 0–3) and high expression (score 4–9) according to the receiver operator characteristic (ROC) curve. The expression of CXCR4 and β-arrestin2 was evaluated similarly.

### RNA extraction and real-time quantitative PCR (RT-qPCR)

Total RNA from fresh-frozen tissue specimens or cell lines was extracted using TRIzol reagent (Invitrogen) according to the manufacturer's instructions. Followed by RT-qPCR as described previously [6], using GAPDH as an internal standard. The sequences of primers used for RT-qPCR were as followed:

USP33: 5′-TGTGATGCTTAGGCAAGGAG-3′, 5′-GGCCCTCCACCATAAATAGA-3′ [8], CXCR4: 5′-ATGAAGGAACCCTGTTCCCGT-3′, 5′-AGATGAT GGAGTAGATGGTGGG-3′ [41], GAPDH: 5′-GCCGC ATCTTCTTTTGCGTCGC-3′, 5′- TCCCGTTCTCAG CCTTGACGGT-3′.

### Cell lines and cell culture

Human CRC cell lines (LoVo, SW480, SW620) were purchased from the American Type Culture Collection (ATCC, Manassas, VA, USA). The NCM460 normal colonic epithelial cell line was attained from Jennio Biotechnology (Guangzhou, China). SW480 and SW620 cells were cultured in Dulbecco's modified Eagle's medium (DMEM) supplemented with 10% fetal bovine serum (FBS), 100 U/mL penicillin and 100 μg/mL streptomycin. LoVo and NCM460 cells were maintained in RPMI 1640 medium with 10% FBS, 100 U/mL penicillin and 100 μg/mL streptomycin. All cells were cultured at 37°C with 5% CO_2_.

### Western blot

A Western Blot assay was performed to measure the protein levels in tissues and cells. Briefly, tissues and cells were lysed and homogenized using RIPA buffer or NP-40 lysis buffer containing protease inhibitor cocktail, phosphatase inhibitor cocktail, and *N*-ethylmaleimide (NEM). The total protein concentration was evaluated using Pierce BCA Protein Assay Kit (Pierce), and about 20 μg proteins were loaded and separated by SDS-PAGE gels. The proteins were then electrotransfered to a PVDF membrane. Membranes were blocked with bovine serum albumin (BSA) and incubated with primary antibodies. The immunoreactivity was amplified by HRP conjugated secondary antibodies and developed with autoradiographic film (Thermo Fisher Scientific). Images were scanned with an EPSON Perfection V37 scanner and semi-quantified with ImageJ software (NIH).

### DNA constructs, siRNA and transfection

HA-ubiquitin construct was purchased from Addgene (Plasmid #18712). Human Flag-HA-USP33 plasmids was also purchased from Addgene (Plasmid #22601), followed by the depletion of Flag-tag to obtain HA-USP33 construct. The inactive USP33 mutant HA-USP33^cys:his^ (C214S/H683Q) [11] was then introduced by site-directed mutagenesis. Flag-β-arrestin2 construct was constructed as described previously [12]. All of the DNA constructs were verified by DNA sequencing before transfection.

USP33 or CXCR4 expression was silenced in LoVo cells using specific small interfering RNA (siRNA) duplex. The USP33 target sequences were 5′- CAAUGUUAAUUCAGGAUGA-3′, the CXCR4 target sequences were 5′-UACUUGUCCGUCAUGCUUCUU-3′ [42], and the negative-control siRNA (NC-siRNA) sequences were 5′-UUCUCCGAACGUGUCACGU-3′. All the siRNAs were synthesized by Shanghai Gene Pharma (Shanghai, China).

Cells were transfected by Lipofectamine 2000 (Invitrogen) according to the manufacturer's instructions.

### Wound-healing assay

LoVo cells transfected with different plasmids or siRNAs were cultured to full confluence in 6-well plates and subsequently scratched using sterile pipette tips. After scratching, the wells were gently washed with RPMI 1640 to remove the detached cells, then added the medium containing SDF-1 (100 ng/mL). Scratched cells were photographed under an inverted microscope after 0, 8, 12, and 24 hours. Migration of cells was evaluated by measuring the width of the scratched area at each time point from at least three separate experiments.

### Proliferation assay

MTT (3-[4, 5-dimethylthiazol-2-yl]-2, 5-diphenyl-tetrazolium) assay was carried out to evaluate the proliferative capacity of USP33-overexpression or USP33-silencing cell lines. Forty-eight hours after transfection, the cells were seeded into a 96-well plate at a density of 3,000 cells/well and cultured in RPMI 1640 containing SDF-1 (100 ng/mL). At designated time points (12, 24, 36, 48, 60, and 72 hours), 20 μL of 5 mg/mL MTT was added into the wells, and incubated for another 4 hours, then removed the medium, and 150 μL DMSO was added to fully solubilize the MTT. The absorbance values were measured at 570 nm wavelength and normalized with cell numbers. Each experiment was performed in three parallel wells and repeated at least three times.

### Invasion assay

Approximately 2 × 10^5^ cells/mL of LoVo cells were seeded in the upper chamber of BD Matrigel Invasion Inserts (BD Biosciences) for 8 h culture in full RPMI 1640 medium for cell adhesion. This was replaced with serum-free medium containing 100 ng/mL SDF-1, while medium with both 10% FBS and 100 ng/mL SDF-1 wad added to the lower chamber. Twenty-four hours after treatment, membranes were fixed with paraformaldehyde (PFA) according to manufacturer's instructions, followed by staining with crystal violet. Cells on the bottom of the membrane were counted using a light microscope. The number of invading cells was quantified from five randomly selected visual fields. All experiments were performed at least three times.

### Immunoprecipitation

The immunoprecipitation experiment was performed to identify the complex formation between USP33 and β-arrestin2. In detail, HA-USP33 plasmids were transfected into LoVo cells with or without CXCR4-knock-down.

Twenty-four hours after transfection, the cells were starved overnight and stimulated with SDF-1 (100 ng/mL) for 15 min. Then cells were harvested and anti-HA beads were added into cell lysates for immunoprecipitation overnight at 4 °C. The existence of protein complexes was detected by Western Blot with specific antibodies.

### Ubiquitination assay

LoVo cells were co-transfected with Flag-arrestin2 and HA-ubiquitin for 48 h, followed by starvation for 4 h. Cells were then stimulated with SDF-1 (100 ng/mL) for 1 h and lysed, immunoprecipitation was then performed with Flag-beads and HA-beads, respectively. After binding overnight, the beads were spun down, washed three times, and SDS-page loading buffer was added to denature the proteins. The ubiquitinated proteins were then analyzed with Western Blot as described above.

### Cell surface ELISA

Cell surface ELISA was performed to measure the internalization and recycling of CXCR4.

For internalization measurement, LoVo cells were seeded into poly-L-lysinecoated 24-well plates (Sigma-Aldrich) 24 h after transfection. Cells were cultured for another 24 h, after which the cells were starved for 4 h and then stimulated with SDF-1 (100 ng/mL) for 30 min and 60 min, respectively. Medium was removed, and cells were washed with ice-cold PBS, followed by cell fixation with 4% PFA/TBS for 10 min on ice. Fixed cells were washed with TBS three times, blocked with 5% BSA/TBS, followed by 1 h incubation with CXCR4 primary antibody (1:500) and another 45 min incubation with goat anti-rabbit alkaline phosphatase-conjugated antibody (1:1000, Sigma). Cells were then washed with TBS three times and incubated with alkaline phosphatase substrate in diethanolamine buffer (Bio-Rad Laboratories) for 15 min at 37°C. Reaction was stopped by adding NaOH (0.4M) into the wells, and a 100 μL-aliquot was used to measure the absorbance at 405 nm wavelength.

For the evaluation of CXCR4 recycling, a similar procedure was carried out as published elsewhere [33, 43]. After starvation, cells were washed with PBS containing Ca^2+^, Mg^2+^, and then incubated with CXCR4 primary antibody in the same PBS buffer for 1 h on ice to label the receptors on cell surface. Cells were then washed and stimulated with SDF-1 (100 ng/mL) for 1 h at 37°C. Following, cells were quickly washed with PBS containing 0.04% EDTA (without Ca^2+^ or Mg^2+^) to remove un-internalized surface receptor-binding antibody and then incubated with AMD3100 (10 μM, CXCR4 antagonist, to block any further internalization) for another 1 h at 37°C. Theoretically, only the receptors that recycled back to the cell surface possess the interaction with primary antibody, thus can be detected using cell surface ELISA method as described above.

### Statistical analysis

The overall survival (OS) was defined as the period from liver metastases resection to the date of death or the last follow-up; and the disease-free survival (DFS) was measured from the date of liver metastases resection to the date of recurrence or the date of death or the last day of follow-up.

All of the statistical data was calculated by SPSS 20.0 software (IBM). Continuous variables were analyzed with Spearman's rank correlation and Student's *t-test*, while categorical variables were analyzed using the χ^2^ test. The Kaplan-Meier method and log-rank test were used to evaluate the prognostic value of USP33 expression and clinicopathological patient characteristics. Both univariate and multivariate Cox proportional hazard regression models were used to identify the independent prognostic factors associated with OS or DFS. Only the variables significantly associated with OS or DFS by univariate analysis were used as covariates for the multivariate analysis. *P* < 0.05 was considered as statistical significant.

## CONCLUSIONS

In summary, our data identified USP33 as a novel prognosis factor for advanced patients with CRCLM. The tumor suppressing role of USP33 is at least partially dependent on β-arrestin-mediated ERK signaling downstream of SDF-1/CXCR4, demonstrating the prospect of deubiquitinating enzyme modulator and biased-ligand development of GPCRs in cancer therapy.

## SUPPLEMENTARY MATERIALS


