# Supplementary material for: Prognostic significance of USP33 in advanced colorectal cancer patients: new insights into β-arrestin-dependent ERK signaling
Source: Oncotarget. 2016 Nov 8;7(49):81223–40. doi: 10.18632/oncotarget.13219 (PMC5348388; doi:10.18632/oncotarget.13219)
Supplement: Supplementary file 1 [file oncotarget-07-81223-s001.pdf]

Prognostic significance of USP33 in advanced colorectal cancer patients: new insights into β-arrestin-dependent ERK signaling

Supplementary Materials

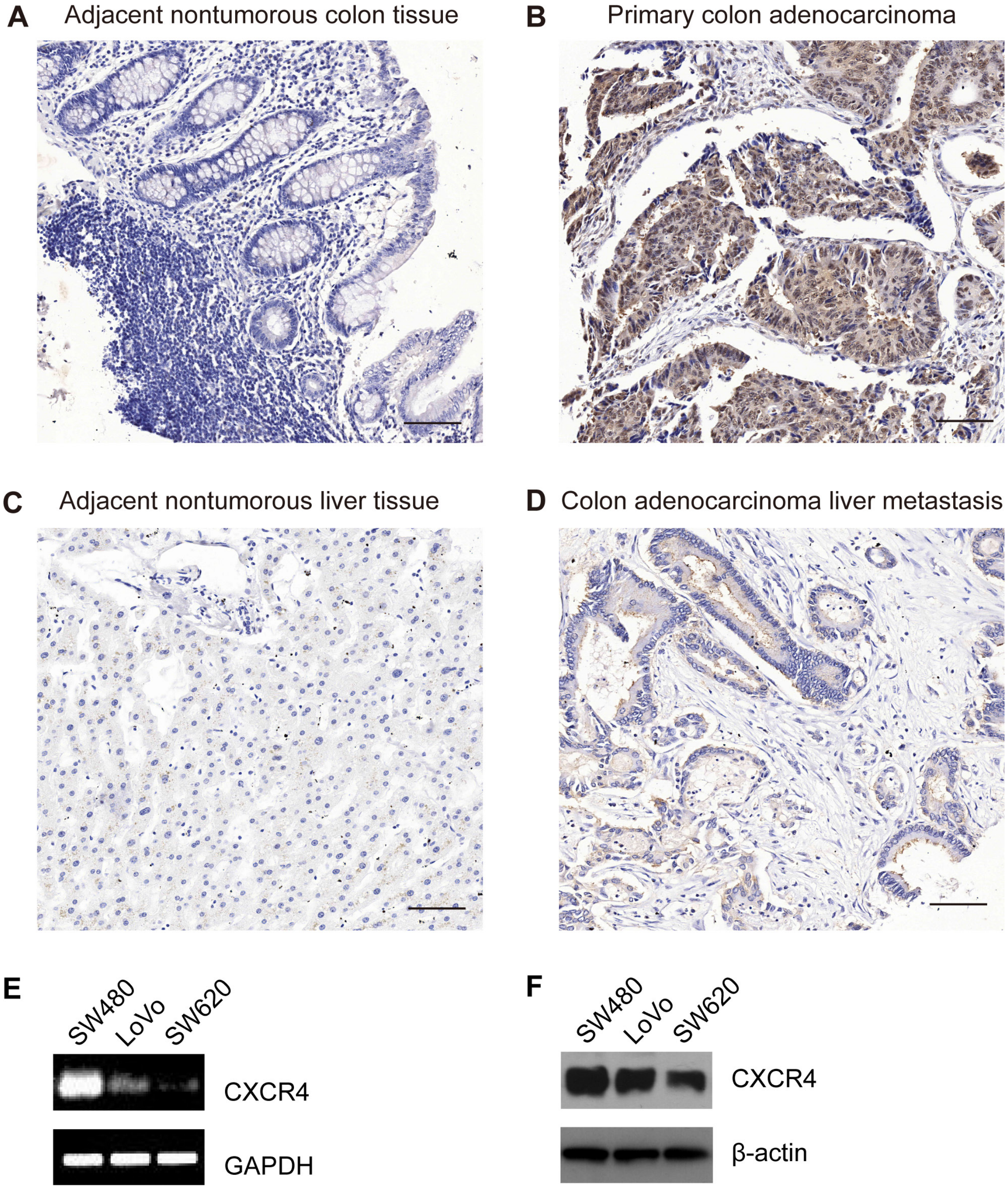

Supplementary Figure S1: Expression pattern of CXCR4 in clinical specimens and cell lines. Representative immunostaining of CXCR4 expression in adjacent non-tumorous colon tissue (**A**, low expression), primary CRC tissue (**B**, high expression), adjacent nontumorous liver tissue (**C**, low expression), and CRCLM tissue (**D**, high expression) by IHC analysis. (**E**) The mRNA expression level of CXCR4 was determined by RT-PCR. SW480 cells showed the highest CXCR4 expression, while SW620 cells had the lowest expression. (**F**) Western Blot assay was performed to measure CXCR4 protein expression in the three different cell lines. The LoVo cells showed moderate expression compared with the other two cell lines, which is consistent with the mRNA level. (scale bar: 100 μM)

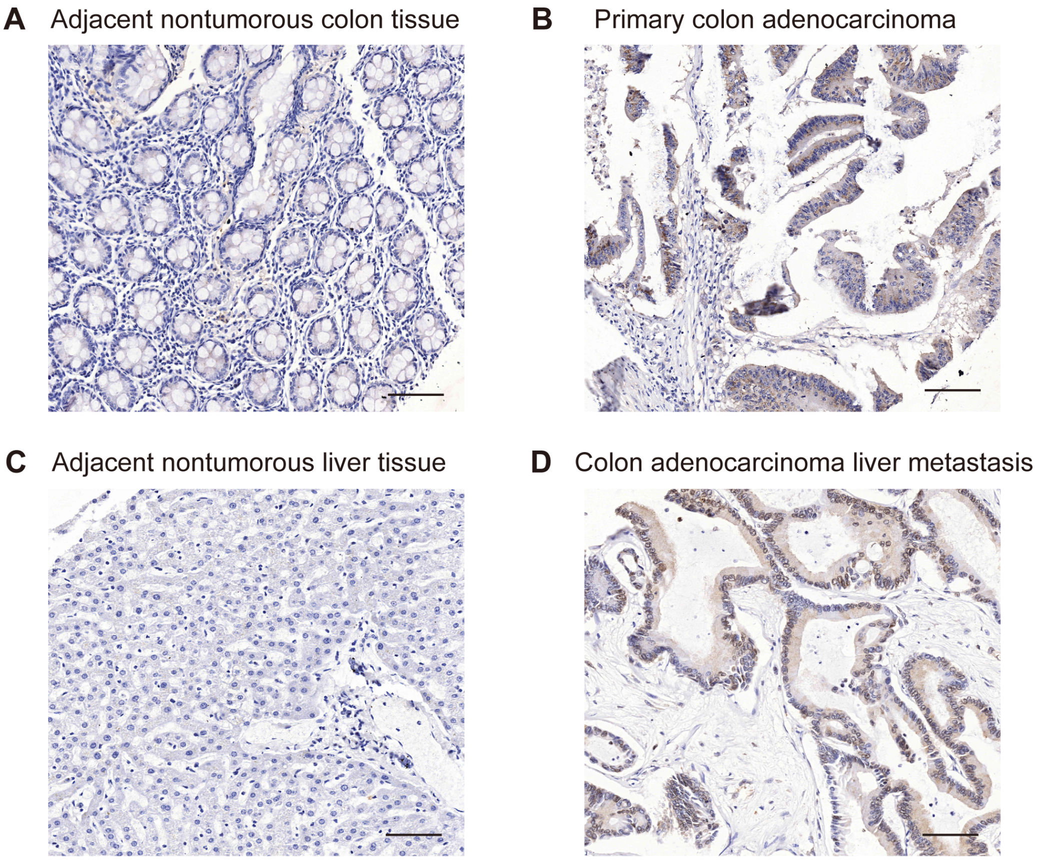

E Expression in primary colon adenocarcinoma

		β-arrestin2			
		High	Low	Total	P value
USP33	High	19	34	53	0.690
	Low	28	58	86	
	Total	47	92	139	

F Expression in colon adenocarcinoma liver metastasis

		β-arrestin2			
		High	Low	Total	P value
USP33	High	18	67	85	0.909
	Low	11	43	54	
	Total	29	110	139	

Supplementary Figure S2: Expression of β-arrestin2 in clinical specimens and its correlation with USP33. Representative immunostaining of β-arrestin2 expression in adjacent non-tumorous colon tissue (**A**, low expression), primary CRC tissue (**B**, high expression), adjacent non-tumorous liver tissue (**C**, low expression), and CRCLM tissue (**D**, high expression) by IHC analysis. (**E**) The relationship between USP33 and β-arrestin2 expression in primary CRC tissues was determined by chi-square test, which showed no statistical significance. (**F**) The relationship between USP33 and β-arrestin2 expression in CRC liver metastases tissues was determined by chi-square test, showing no significant correlation. (scale bar: 100 μM).

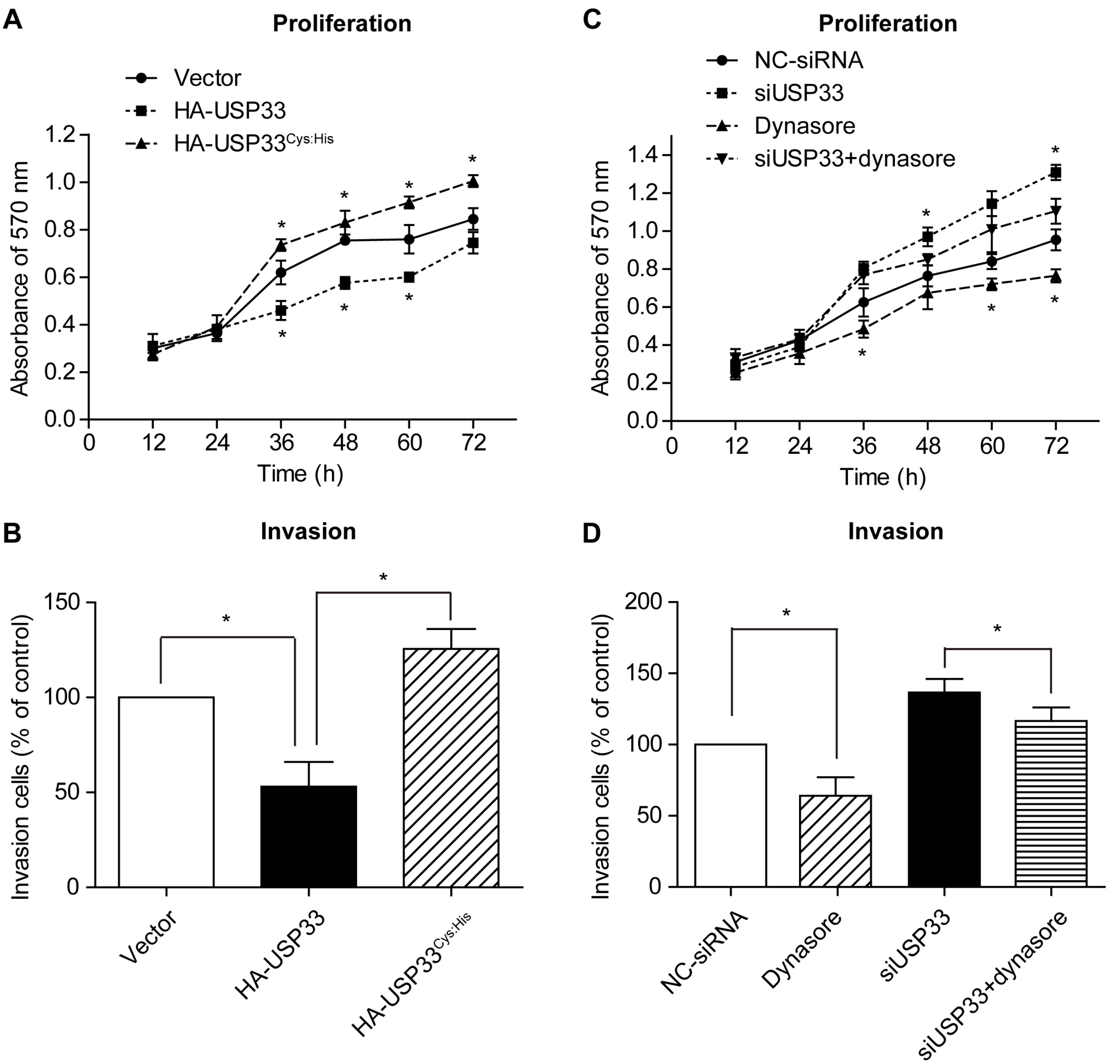

Supplementary Figure S3: Inhibiting effects of USP33 in cell proliferation and invasion function through regulating internalization-mediated ERK signaling and are dependent on its enzymatic activity. Overexpression of USP33Cys:His, the inactive mutant, can increase cell proliferation (**A**) and invasion (**B**), which was completely different from the wild type USP33. The reverse effects of the mutant may result from its competitive inhibition with endogenous USP33, indicating the enzymatic activity of USP33 is critical for its tumor suppressing effects. (**C, D**) The internalization inhibitor, dynasore, can slightly decrease the tumorigenic prevalence of LoVo cells. The attenuate effects of dynasore implicated that the tumor suppressive function of USP33 is at least partially dependent on inhibiting the internalization-dependent "late" ERK signaling. The results are the mean ± S.D. from three independent experiments (*$P < 0.05$).

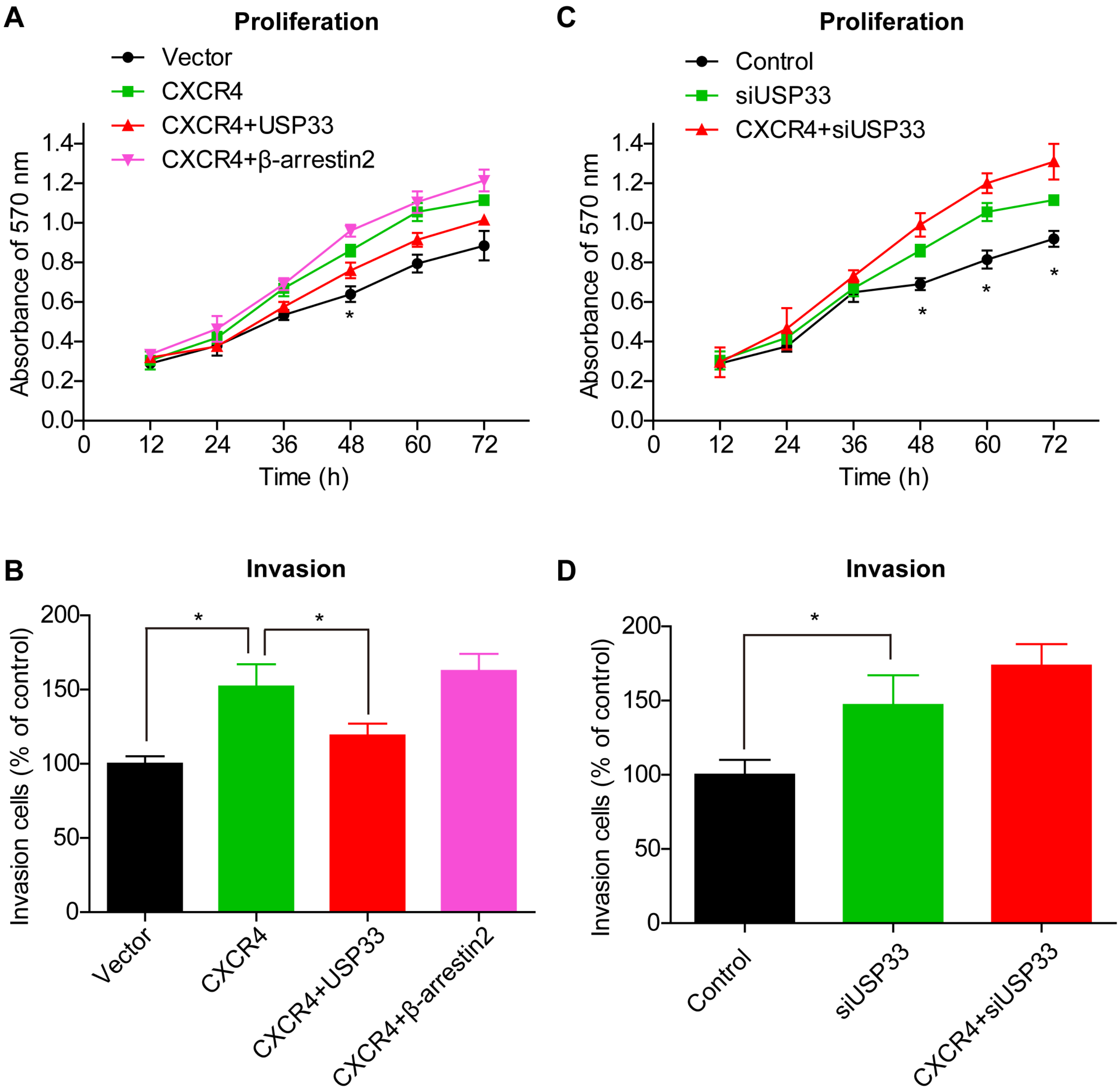

Supplementary Figure S4: Effects of USP33 in regulating cell proliferation and invasion in CXCR4-overexpressed cells. (**A, B**) Overexpression of CXCR4 can significantly promote the proliferation and invasion of LoVo cells. Co-expression of β-arrestin2 can enhanced cells' carcinogenicity; while co-expression of USP33 showed opposite effects. (**C, D**) In the USP33-silencing LoVo cells, overexpression of CXCR4 can synergistically enhance the tumor-promoting effects of USP33-siRNA. The results are the mean ± S.D. from three independent experiments (*$P < 0.05$).
